# A Microbiomic Analysis of a Pasture-Raised Broiler Flock Elucidates Foodborne Pathogen Ecology Along the Farm-To-Fork Continuum

**DOI:** 10.3389/fvets.2019.00260

**Published:** 2019-08-07

**Authors:** Michael J. Rothrock, Aude Locatelli, Kristina M. Feye, Andrew J. Caudill, Jean Guard, Kelli Hiett, Steven C. Ricke

**Affiliations:** ^1^Egg Safety and Quality Research Unit, U. S. National Poultry Research Center, United States Department of Agriculture - Agricultural Research Service (USDA-ARS), Athens, GA, United States; ^2^Department of Food Science, Center for Food Safety, University of Arkansas, Fayetteville, AR, United States; ^3^Department of Public Health, University of Georgia, Athens, GA, United States; ^4^Office of Applied Research and Safety Assessment, Center for Food Safety and Applied Nutrition, Food and Drug Administration (FDA), Laurel, MD, United States

**Keywords:** microbiome, pastured poultry, *Salmonella*, *Campylobacter*, ecology

## Abstract

While conventionally grown poultry continues to dominate the U. S. poultry industry, there is an increasing demand for locally-grown, “all natural” alternatives. The use of next generation sequencing allows for not only the gross (e.g., community structure) but also fine-scale (e.g., taxa abundances) examination of these complex microbial communities. This data provides a better understanding of how a pasture flock's microbiome changes throughout the production life cycle and how that change in microbial ecology changes foodborne pathogens in alternative poultry production systems. In order to understand this ecology better, pooled broiler samples were taken during the entire flock life cycle, from pre-hatch gastrointestinal samples (*N* = 12) to fecal samples from the brood (*N* = 5), and pasture (*N* = 10) periods. Additional samples were taken during processing, including skin and feather rinsates (*N* = 12), ceca (*N* = 12), and whole carcass rinses (*N* = 12), and finally whole carcasss rinsates of final products (*N* = 3). Genomic DNA was extracted, 16S rDNA microbiome sequencing was conducted (Illumina MiSeq), and microbiomes were analyzed and compared using QIIME 1.9.1 to determine how microbiomes shifted throughout production continuum, as well as what environmental factors may be influencing these shifts. Significant microbiome shifts occurred during the life cycle of the pasture broiler flock, with the brood and pasture fecal samples and cecal samples being very distinct from the other pre-hatch, processing, and final product samples. Throughout these varied microbiomes, there was a stable core microbiome containing 13 taxa. Within this core microbiome, five taxa represented known foodborne pathogens (*Salmonella, Campylobacter*) or potential/emerging pathogens (*Pseudomonas, Enterococcus, Acinetobacter*) whose relative abundances varied throughout the farm-to-fork continuum, although all were more prevalent in the fecal samples. Additionally, of the 25 physiochemical and nutrient variables measured from the fecal samples, the carbon to nitrogen ratio was one of the most significant variables to warrant further investigations because it impacted both general fecal microbial ecology and *Campylobacter* and *Enterococcus* taxa within the core fecal microbiomes. These findings demonstrate the need for further longitudinal, farm-to-fork studies to understand the ecology of the microbial ecology of pasture production flocks to improve animal, environmental, and public health.

## Introduction

The gastrointestinal tract (GIT) of poultry hosts a complex and dynamic bacterial microbiota (1), and these microbial communities can directly affect animal, environmental, and public health (2, 3). Studies have shown that environmental factors such as hatchery hygiene levels (4), housing (5), and production system (6, 7), litter quality and management (8, 9), and climate and geographical locations (10, 11) can significantly influence poultry GIT microbiota and the diversity demonstrate the dynamics of GIT microbial ecology. Additionally, the poultry GIT microbiome can serve as a reservoir for zoonotic pathogens like *Campylobacter, Salmonella*, and *Acinetobacter* spp. (12). Therefore, investigations into the dynamics of poultry microbiomes are understood throughout the entire farm-to-fork continuum.

Early in the poultry production chain, the colonization of the GIT of newly hatched chicks is a combination of the hen and hatchery environment during the pre-hatch phase (13). The GIT microbiome diversity of the very young chick (0–1 weeks old) increases gradually and with significant population variability compared to older mature birds (14), even within the same farm or flock (15, 16). While zoonotic pathogen colonization can occur at any stage of the farm-to-fork continuum, the lack of a mature GIT microbial ecology makes newly-hatched chicks susceptible (17). The source of these pathogens that colonize juvenile birds are not only from the surrounding farm environment (18, 19), but also from the other chickens within a flock (20, 21). These observations suggest that the environmental influences that drive the GIT microbiome diversification and establishment of these birds early in life have a lasting effect throughout the pre-harvest and grow-out periods. The poultry microbiome and resident zoonotic pathogens such as *Salmonella* and *Campylobacter* can be transferred from the farm pre-harvest environment to the post-harvest processing environment, and ultimately, the consumer (22–24). Therefore, it is important to attempt to understand these longitudinal dynamics from farm-to-fork.

A recent study attempted to take a farm-to-fork approach to poultry (23); however, there was no direct link between the pre-harvest, post-harvest, and final product samples analyzed, and the focus was on conventionally grown poultry. While conventionally grown chicken account for the majority of the poultry products produced in the U.S., consumers are increasingly concerned with the safety and welfare of poultry produced within conventional systems (25–27). This has resulted in an increased commercial demand for alternatively grown poultry products (28). Within the state of Georgia, which is the largest conventional poultry producing state in the U.S., 97% of the respondents of an online survey stated that they were very supportive of organic or all-natural poultry products that are locally grown on small farms. Furthermore, respondents would consider considering shifting their poultry purchases from conventional sources even when prices for pasture flocks reached $5.00 a pound (29). One alternative production system that is growing in popularity is pasture-raised poultry, which requires flocks to have continual access to fresh pasture and the outdoor environment on a daily basis (27, 30, 31). There is a limited amount of research available regarding the overall microbial community and the resulting foodborne pathogen dynamics within this production system [see (32) for a recent review].

Therefore, to better understand the dynamics of general microbial populations and foodborne pathogens within GIT communities, a single pastured-raised broiler flock was followed throughout the entire production continuum. To accomplish this, samples were collected from the flock during the pre-hatch, pre-harvest (brood, pasture), processing, and to the final product. Then, 16S rDNA microbiome sequencing was performed using the Illumina MiSeq platform. The data was analyzed with QIIME and comparisons were made between the microbiomes of various sample types (GIT, feces, ceca, carcass rinses) and stages along the farm-to-fork continuum. By comparing these microbiomes within sample type, not only among sample types and stages, but also to physiochemical data collected during the pre-harvest live production period, environmental influences of these general and pathogenic communities could be potentially elucidated, which could be used to better understand the drivers of these bacterial community throughout the broiler's life before reaching the consumer.

## Materials and Methods

### Hatchery Sample Collection

A commercial broiler hatchery in the southeastern U.S. provided all of the eggs for this study. The broilers used for this study were a Cobb 500 cross. Once the eggs were set in the commercial hatchery, eggs (*n* = 25 total) were collected at four time points: (1) 1 week after set, (2) 2 weeks after set, (3) after *in ovo* immunization (2.5 weeks after set), and (4) one-day post-hatch. All necropsies throughout the course of the pre-hatch component of the study were performed at the University of Georgia Poultry Disease and Research Center (Athens, GA, USA) and all work was covered under Institutional Animal Care and Use Committee (IACUC) number A2010 11-568-Y1-A0.

At each sampling time, necropsies were performed to aseptically remove the embryonic gastrointestinal tracts (GIT) from each egg. Eggs were removed from the 37°C incubator, placed in a Type II biosafety cabinet (BSC), sprayed with 0.4% Bioguard (Neogen Corp, Lansing MI, USA), and allowed to dry prior to sampling. Once the embryos were dry, sterile forceps were used to crack the egg at the air cell end. The egg shell was discarded, the embryos removed from the shell with sterile forceps and the embryos were euthanized by cervical dislocation (CD). Embryos were pooled in groups of seven into a sterile 110 mm^3^ petri dish and sampled. The abdominal cavity of the embryos was opened with sterile scissors and the intestines were removed with sterile forceps. The GIT samples from each group of seven embryos were placed into a small filtered stomacher bag (Seward Laboratory Systems, Inc., Davie, FL).

For the post-hatch sample collection, an extra set of eggs were collected from the commercial hatchery, the eggs were placed into hatching baskets by breeder flock, and were then set in a single stage Natureform Hatcher (NatureForm Hatchery Technologies, Jacksonville, FL) and allowed to hatch at the University of Georgia facility. Chicks were removed from the hatcher, placed in ventilated transport containers and transported to the lab. For each group, chicks were euthanized by CD and placed on sterile 110 mm^3^ petri dishes inside the BSC. The GIT samples were collected and pooled as described above.

Each pooled GIT sample was weighed and sterile 1x phosphate-buffered saline (PBS) was added to pooled GIT samples (3:1; 1x PBS volume: GIT mass) to ensure enough homogenate was available for all analytical needs. The pooled GIT samples were homogenized via stomaching (Seward Laboratory Systems, Inc.) on max speed for 60 s. Two 0.5 mL aliquots per sample were placed into separate FastPrep Lysing Matrix A tubes (MP Biomedicals, Solon, OH, USA), and all tubes were then frozen at −20°C until DNA extraction.

### Brood and Pasture Sample Collection

After the post-hatch GIT samples were collected, a set of 50 1-day old chicks were transported in chick carriers to a small pastured poultry homesteading farm ~3 acres in size in north-central Georgia. The facility collectively rears the broilers with pastured layer hens, pastured guinea hens, dairy goats, a small flock of sheep, as well as housing a small swine herd on an adjacent, but completely separate, plot of land. The swine herd and sheep flock had <5 animals throughout the course of the study. While the above animals were grown for agricultural purposes, the homesteading farm also housed one horse, one cow, and one goat within the same pasture during pastured broiler live production.

Chicks were brooded through 3 weeks of age in two groups of 25 chicks housed within separate 80-gallon plastic totes with wood chip bedding. Chicks were given food and water *ad libitum*, and fresh bedding was overlaid over old bedding (deep litter method) every day. The bedding was completely removed and replaced weekly. Since the totes were kept within the farmer's house, no heat lamps were required during the brooding stage. For the first week post-hatch, all accessible fecal samples were aseptically scrapped from the liners at the bottom of the chick carriers and pooled into a single initial fecal sample. Weekly fecal samples were collected from week 1 to week 3 post-hatch, and all observable fresh fecal samples were removed from both of the totes and pooled into a single sample for that sampling point, with care being given to remove as much bedding material as possible from the sample.

By 4 weeks of age, the chickens were moved to mobile pens on the farm pasture. The mobile pens house 25 birds per pen, had a foot print of ~72 ft^2^ (6 × 12 ft), and contained a waterer, feeder, and roosting bars. The mobile pens were covered by plastic tarps to provide some environmental protection, these pens were moved daily to fresh pasture, and during the day the broilers were given access to pasture outside of the pen. Birds were fed and watered *ad libitum* and were not physically handled unless necessary for their safety or protection. The birds were grown this way on pasture until 16 weeks of age. Through week 8, fresh fecal samples were collected on a weekly basis. After week 8, sampling occurred every other week until 16 weeks of age when the birds were processed. For fecal sampling, after the mobile pens were moved for the day, all fresh fecal samples from the previous mobile pen area were collected and pooled into a single broiler fecal sample for that time point. During sampling, any fecal samples that could be identified as belonging to another animal species on the farm (horse, cow, goat, layer, guinea hen) within the area the broilers were currently being reared were also collected and processed in the same manner as the broiler feces, described below.

For all fecal samples, pooled fecal samples were placed on ice at the farm and transported back to the laboratory. Pooled fecal samples were weighed into three separate 0.5 g subsamples, and each of these subsamples were placed into separate FastPrep Lysing Matrix E tubes (MP Biomedicals), and all tubes were then frozen at −20°C until DNA extraction.

### Processing and Final Product Sample Collection

At 16 weeks of age, after a 24-h feed withdrawal, the broilers were moved individually to the processing area on the farm. Broilers were culled via exsanguination using “kill cones,” and post-bleed out the head, feet, and wing tips were removed. The farmer completely removed the skin and feathers from the carcass, and then the entire viscera was subsequently removed. Removed skin with feathers were placed into individual sterile plastic bags containing 250 mL of 10 mM PBS and shaken vigorously manually for 1 min to produce a skin with feather rinse (SFR) sample. The rinsate was then poured into a filtered stomaching bags (Seward Laboratory Systems, Inc., Davie, FL). For each carcass, ceca were aseptically removed at the cecal tonsil juncture and placed into sterile, filtered stomaching bags.

Carcasses were rinsed using non-chlorinated well water and placed on ice until all carcasses were processed, which acted as the chilling step. The average time from kill cone to chilling was 12 min per bird per farmer, so with two farmers processing birds, the entire flock was processed in ~5 h. The processed and chilled carcasses were moved into the house and rinsed internally and externally with in a dilute vinegar solution. The next step is termed the post-processing whole carcass rinse (P-WCR). For these sample collections, chilled carcasses were placed into individual sterile plastic bags containing 100 mL 10 mM PBS and shaken vigorously manually for 1 min, with the resulting rinsate being placed in sterile filtered stomaching bags and stored on ice for transportation to the lab. The carcasses were then wrapped using food grade plastic wrap and placed at 4°C for 24-h. At that time, the carcass was considered the final product that the farmer provides to the customers using a customer-supported agriculture (CSA) model. Final product whole carcass rinse (FP-WCR) samples were created using the protocol described above for the P-WCR samples on those carcasses.

All SFR, cecal, P-WCR, and FP-WCR samples were transported back to the lab on ice and processed within 2 h post-collection. Cecal samples were diluted 1:3 using 10 mM PBS, while no buffer addition was needed for the three rinsate samples. All samples were homogenized for 60 s at the maximum setting and 0.5 mL of each sample was placed into separate FastPrep Lysing Matrix E tubes (MP Biomedicals), and all tubes were subsequently frozen at −20°C until DNA extraction.

### DNA Extraction, Microbiome Sequencing, and Data Analysis

Genomic DNA was extracted from the GIT, feces, ceca, SFR, P-WCR, and FP-WCR samples using a hybrid extraction method optimized for poultry samples (33). In short, 1 mL of Qiagen ASL buffer (Qiagen, Valencia, CA, USA) was added to each Lysing Matrix sample tube and vortexed at the maximum setting for 1 min, followed by a more thorough homogenization using the FastPrep 24 (MP Biomedicals) at 6.0 m/s for 45 s. After centrifugation (14,000° g for 10 min), supernatant was removed, added to a sterile 2 mL tube, and incubated at 95°C in a water bath for 5 min. At this point, all samples were processed using the QIAamp DNA Stool Mini Kit (Qiagen, Hilden, Germany) using the QIAcube robotic workstation (Qiagen) and the stool pathogen detection protocol. After the automated extraction and purification steps, the two extracted aliquots for each pooled sample were combined in 100 mL sterile molecular grade water using Vacufuge^TM^ Plus (Eppendorf, Hauppage NY, USA), and the DNA concentration in each sample was determined spectrophotometrically using the Take3® plate in conjunction with the Synergy H4 multimode plate reader (BioTek, Winooski, VT, USA).

Library construction and sequencing were performed by the Earth Microbiome Project Laboratory at the U.S. Department of Energy, Argonne National Laboratory (Argonne, IL). In short, the hypervariable V4 domain of bacterial 16S rDNA gene was amplified using the F515 (5′-CACGGTCGKCGGCGCCATT-3′) and R806 (5′-GGACTACHVGGGTWTCT AAT-3′) primer set with each primer containing Illumina adapter sequences (Illumina, Inc., San Diego, CA) and the reverse primer containing the Golay barcodes to facilitate multiplexing (34). Raw reads were obtained by using the Illumina MiSeq platform.

A total of 17,700,915 raw reads were generated and processed by the QIIME v1.9.1 (Quantitative Insights Into Microbial Ecology) pipeline (35). Forward and reverse sequence reads were merged according to the fastq-join parameter within the *join_paired_ends.py* command. Quality filtering and library splitting according to the Golay barcode sequences were performed on the merged sequences with *split_library_fastq.py* script (-q 19, all other parameters were default) and resulted in a total of 13,419,288 sequences with an average of 74,139 sequences per sample. Sequences were chimera checked against the Greengenes 13_8 database (36) and clustered into Operational Taxonomic Units (OTUs) according to their sequence similarity (97%) using the usearch option (37) with *pick_otus.py* script (-m usearch, all other parameters were default). A representative sequence for each OTU was selected with *pick_rep_set.py* script (default parameters) and used for taxonomic assignment using UCLUST and the Greengenes 13_8 database (36) with *assign_taxonomy.py* (default parameters). Sequences were aligned (*align_seqs.py script*, default parameters) using PyNAST (38) and filtered (*filter_alignment.py*, default parameters). A phylogenetic tree was subsequently produced with the *make_phylogeny.py* script (with default settings and FastTree program). This pipeline resulted in a total of 1,106,557 sequences were obtained with an average of 52,693 sequences per sample for further analysis. Overall, a total of 1,789 unique OTUs were identified across all samples. The raw sequence and metadata files have been deposited in the MG Rast public database and is accessible with the MG-Rast ID mgm4844877.3.

Alpha diversity was used to describe the microbial richness, evenness and diversity within samples using the Chao1, Equitability, and Shannon metrics. Significant differences in alpha diversity parameters were tested between the sample types and different stages using the *compare alpha diversity.py* script. Beta diversity was determined using the Bray-Curtis distance to measure the dissimilarity between samples. Principal coordinate analysis (PCoA) of the Bray-Curtis distance was performed to determine the change in the community structure using the vegan package v2.3-0 (39) in R software v3.2.1. Whole bacterial community composition was examined using non-metric multidimensional scaling (NMDS) of Bray-Curtis dissimilarities with the *metaMDS* function. The function *envfit* was used to calculate the regression statistic for fecal physiochemical variables on ordination scores at a *p* ≤ 0.05. Two different non-parametric analysis methods including analysis of similarities (ANOSIM) and permutation multivariate analysis of variance (PERMANOVA) were used to examine whether there were significant differences in community structures between the different sample types collected throughout the study and also between the different stages of the farm-to-fork continuum. The Bray-Curtis distance was used for the ANOSIM and PERMANOVA analyses in QIIME using *compare_category.py*. Core microbiome analyses were performed using the *compute_core_microbiome.py script* using and minimum fraction for core score of 0.75 (OTU must be in at least 75% of samples).

Using qPCR, total bacteria [16S rDNA gene; (40), *Salmonella* spp. (*invA*) (41), *Campylobacter jejuni* (*hipO*) (42), and *Listeria monocytogenes* (*hylA*) (43)]. All DNA extractions analyzed with qPCR were performed on Mastercycler® ep Realplex s2 and s4 thermocycling machines (Eppendorf) in 20 μL reaction mixture was prepared using 10 μL of 2x PerfeCTa qPCR ToughMix, ROX (Quanta BioSciences, Gaithersburg, MD, USA) and 5 μL template of 1:10 diluted sample (containing 10–15 ng genomic DNA) following the previously published protocols. The PCR amplification efficiency and detection sensitivity were determined by using a series of 10-fold dilutions of standards (10^8^-10^1^ copies per reaction) created from purified plasmids for the target gene. Target gene copy number was determined using Mastercycler ep Realplex software (Eppendorf).

### Fecal Physiochemical Analysis

The moisture content of the fecal samples was determined by drying overnight at 65°C and calculating the difference between the wet and dried weights of the litter. Fecal pH and electrical conductivity (EC) were determined using an Orion Versa Star Advanced Electrochemistry Meter (ThermoScientific) using 1:5 dilutions in distilled water. Fecal samples were submitted to the University of Georgia Soils Testing Laboratory for Total Carbon, Total Nitrogen, and elemental (Al, As, B, Ca, Cd, Cr, Cu, Fe, K, Mg, Mn, Mo, Na, Ni, P, Pb, S, Si, Zn) composition.

## Results and Discussion

### Gross Microbiome Changes Throughout the Farm-To-Fork Continuum

**Figure 1:**
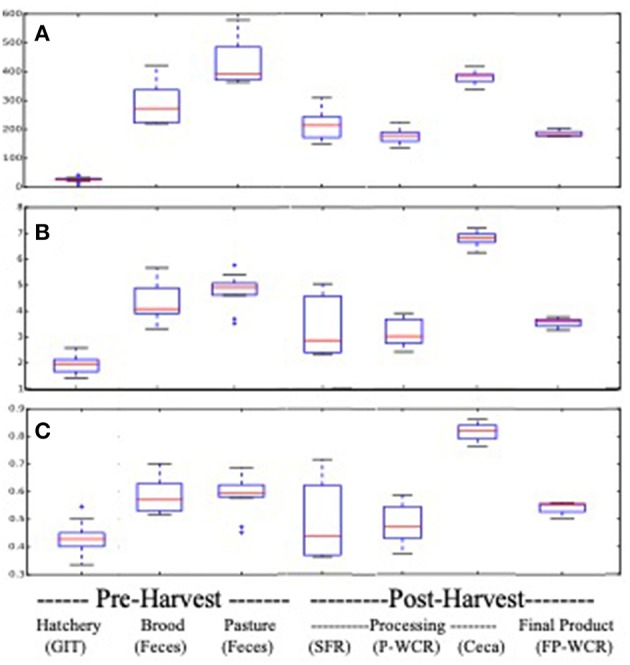
Alpha-diversity boxplots for microbiomes from different sample types and stages along the farm-to-fork continuum of a pasture-raised broiler flock. **(A)** Comparison of richness based on the chao1 metric. **(B)** Comparison of diversity based on the Shannon Diversity metric. **(C)** Comparison of evenness based on the equitability metric.

Pasture flock broiler microbiomes significantly changed throughout the farm-to-fork continuum. Cecal microbiomes possessed the richest, most diverse, and most even communities from all of the assayed samples, although brooder and pasture fecal microbiomes had equivalent richness (Figure 1). Conversely, the hatchery GIT samples possessed the least rich, diverse and even communities. Hatchery GIT samples were significantly (*p* < 0.05) lower than any other sample collected aside from only the FP-WCR sample in terms of evenness. The general trend for all α-diversity estimates was, from highest to lowest, was ceca > pasture feces > brood feces > FP-WCR > SFR > P-WCR > GIT.

**Figure 2:**
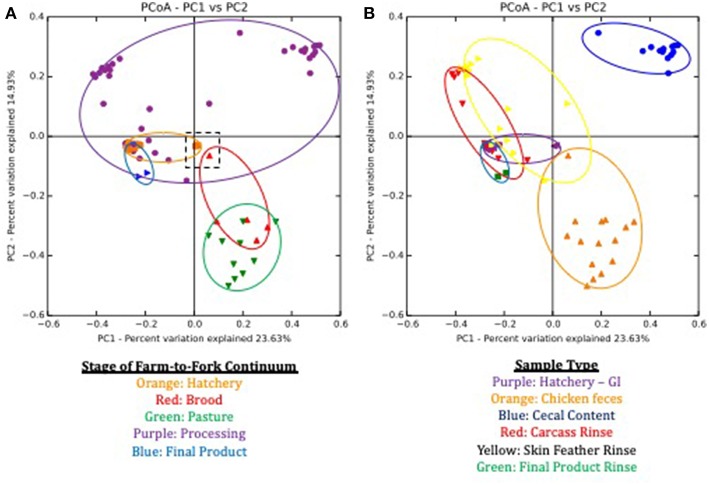
Principle Coordinate Analysis (PCoA) plots based on Bray Curtis dissimilarities of microbiomes during the lifespan of a pasture-raised broiler flock. **(A)** Sample separation based on stage of farm-to-fork continuum, with each stage being assigned a different color. Symbols represent different samples from a given stage, and the ovals encompass the area of the graph that covers all of the samples for a given stage. The dashed black box in the middle of the graph highlights the GIT and feces samples that occur within the first day post-hatch. **(B)** Sample separation based on the sample type, with each sample being assigned a different color. Symbols represent different samples from a given sample type, and the ovals encompass the area of the graph that covers all of the samples for a given sample type.

**Figure 3:**
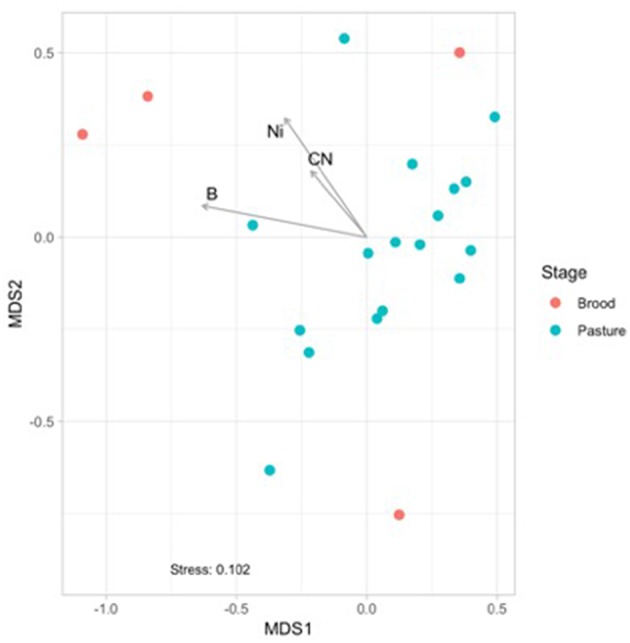
Non-metric multidimensional scaling (NMDS) based on Bray Curtis dissimilarities of broiler fecal microbiomes from the brood and pasture stages. The broiler age (in weeks) is overlaid on the different points within the graph, with 1A−3 representing brood fecal microbiomes and 4A−16 representing pasture fecal microbiomes. Significant physiochemical parameters (B, C:N ratio, Ni; *p* < 0.01) were fitted onto the NMDS plot using the envfit function in the VEGAN package.

Beta-diversity estimates, based on the Bray-Curtis dissimilarity matrix, also showed distinct communities at each stage of the farm-to-fork continuum (Figure 2A) and sample type (Figure 2B). Both farm-to-fork stage (*p* = 0.001; *R*^2^ = 0.675) and sample type (*p* = 0.001; *R*^2^ = 0.391) significantly affected the resulting microbiomes according to ANOSIM analyses. When focusing on the stage of the continuum (Figure 3), microbiomes from all stages were tightly clustered, other than those microbiomes from the processing stage, which encompassed the upper half of the diagram (purple symbols and outline). While there were no major differences between the hatchery and post-harvest (processing, final product) microbiomes in terms of β diversity, the fecal samples (brood, pasture) formed a discrete cluster separate from those samples.

To better understand the large variability within the processing microbiomes, clustering was performed based on sample type (Figure 2B). Sample type clustering showed that the cecal microbiomes (blue symbols and outline) were discretely clustered compared to the SFR (yellow) and P-WCR (red) microbiomes. The two processing rinse sample types clustered closely with the final product rinse (green) and the GIT (purple) microbiomes. These findings are generally consistent with previous studies showing greater diversity and richness in fecal microbiomes compared to post-harvest rinses (23). That data is significantly different microbiomes when comparing pre-harvest (fecal, litter) with intestinal samples (ileum, cecum) both in chickens (44, 45) and turkeys (24), although none of these studies were able to directly link the fecal and post-harvest samples within the same flock as done in this present study.

It is interesting to note the shift in microbiomes between the hatchery and the brooding stage. While the GIT and fecal (brood and pasture) microbiomes generally clustered together, there were a set of outlier samples for both that clustered near each other (Figure 2A, dashed box). The microbiomes in this box represent the GIT samples 1-day post-hatch (orange) and the fecal samples from 1-day old chicks in the brooder box (red). By 1 week of age in the brood box, the fecal microbiomes shift significantly and are clustered with all subsequent fecal microbiomes. There is also a significant shift in total bacterial concentrations in these samples, as assessed by targeted qPCR. One-day post-hatch, the GIT 16S rDNA copy number (5.22 log_10_ copies) significantly increased compared to pre-hatch levels (1.45 log_10_ copies). The 1-day post-hatch fecal samples exhibited a significantly lower 16S rDNA (5.69 log_10_ copies) compared to the rest of the brood or pasture fecal samples (6.98 and 7.34 log_10_ copies). Stable, mature gut microbiomes have been previously shown to develop at various times throughout the broilers' life, ranging from 3 to 6 weeks of age in cecal microbiomes of conventionally-grown broilers (16, 46, 47), but this shift toward a stable microbiome occurred earlier in the present study using the pasture-raised model. This indicates that the shift toward a mature gut microbiome as assessed by fresh feces can be established very early, and this has implications for any attempts to modify or modulate the broiler gut microbiome to improve performance and health through the use of pre- or probiotics, as discussed elsewhere (32, 48–51). This data suggests that application of these products needs to occur immediately post-hatch or potentially even *in ovo* within the hatchery before the stable, mature gut microbiome develops (during the first week of life).

### Potential Environmental or Management Drivers of Fecal Microbiomes

Physiochemical data was collected from the brood and pasture samples to see if they had any potential effects on the fecal microbiomes using non-metric multidimensional scaling (NMDS) analyses (Figure 3). When only considering the fecal samples, there was a separation between the brood (red) and pasture (blue) samples, and three physiochemical parameters were found to be significantly correlated to the brood fecal microbiomes: boron (*p* = 0.048; *R*^2^ = 0.387), nickel (*p* = 0.043; *R*^2^ = 0.554), and carbon to nitrogen (C:N) ratio (*p* = 0.012; *R*^2^ = 0.432). These three variables had no effect on the pasture fecal microbiomes, so it appears that during the first month of life brood microbiomes are significantly influenced by the concentrations of boron, nickel, and the C:N ratio within the feces. This data reinforces that the relatively stable mature gut microbiome is formed early after hatch.

**Figure 4:**
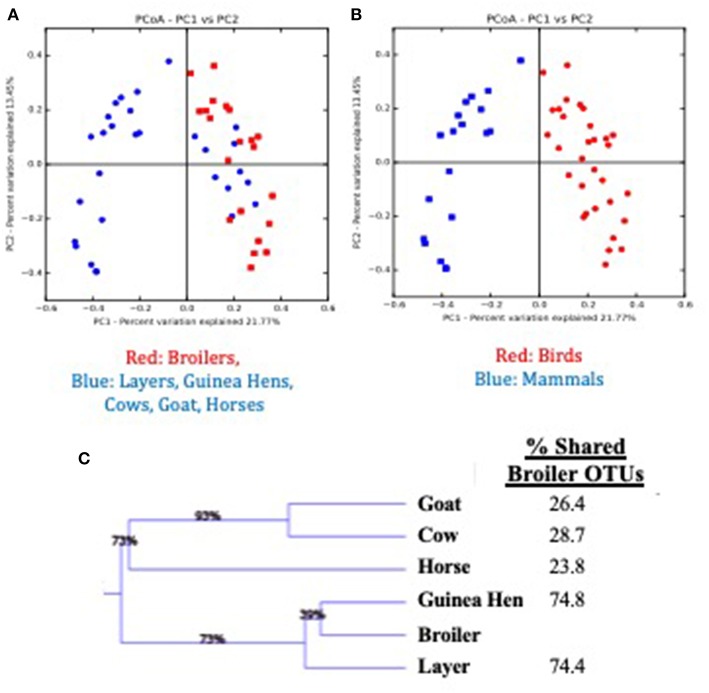
Comparison of feces from multiple animal species present during the pre-harvest (live production) period for a pasture-raised broiler flock. **(A)** Principle Coordinate Analysis (PCoA) plot based on Bray Curtis dissimilarities comparing broiler feces (red) to all other feces (blue) recovered on pasture during live production. **(B)** Principle Coordinate Analysis (PCoA) plot based on Bray Curtis dissimilarities comparing bird feces (Broilers, Layers, Guinea Hens; red symbols) to mammal feces (Cow, Goat, Horse; blue symbols) recovered on pasture during live production. **(C)** WPGMA comparison of fecal microbiomes from different animals, with the final column describing the percent of OTUs shared with the broiler microbiome.

Considering the environmental exposure of these pastured flocks to other animal species on this multi-purpose farm, and the coprophagic nature of broilers, the question arises as to whether the presence of other animals on the farm impact the broilers raised on these pastures. To assess possible broiler microbiome effects, fecal samples from all animals raised on the pasture during the broiler's lifetime (horses, cows, goats, layers, guinea hens) were collected weekly (if present in the current broiler sampling site) and the fecal microbiomes for all animals were compared (Figure 4). When comparing the broilers to all other animals based in Bray-Curtis dissimilarity matrix of β-diversity (Figure 4A), the other animal fecal microbiomes (blue) generally clustered separately from the broiler fecal microbiomes (red), although there was some clustering of non-broiler with the broiler microbiomes. The identities of these similar non-broiler microbiomes were found to be other bird species (layers, guinea hens), with distinct clustering of microbiomes found between bird and mammal species on the farm (Figure 4B; red and blue symbols, respectively). Weighted Pair Group Method with Arithmetic Mean (WPGMA) analyses revealed that mammal fecal microbiomes only shared 24–29% of the OTUs with the broilers, while the other bird species shared ~75% of the OTUs with broiler fecal microbiomes (Figure 4C). While there have been studies that have described the impact that the pasture-raised management model has on biosecurity (52–54) and on the prevalence or abundance of foodborne pathogens (27, 55–57), this data suggests that rearing broilers concomitantly with other mammal species does not significantly affect their gut microbiomes, potentially due to the rapid establishment of a mature broiler gut microbiome.

### Multi-Level Taxonomic Microbiome Changes

There were significant phyla-level differences between the various microbiomes across the farm-to-fork continuum (Table 1). Firmicutes and Proteobacteria accounted for >85% of all OTUs for nearly all sample types, with Firmicutes significantly dominating the brood feces, pasture feces, and the cecal microbiomes and Proteobacteria significantly more abundant in the hatchery, SFR, P-WCR, and FP-WCR communities. Firmicutes and Proteobacteria only account for about 66.5% of the OTUS in the cecal samples, which exhibited significantly higher abundances of Bacteriodetes, Euryarchaeota, and Tenericutes compared to all the other samples collected in the study. The phyla are consistent with other studies and meta-analyses of poultry pre-harvest (23, 33), intestinal (45, 58), and processing (59, 60) microbiomes.

**Table 1:** Relative abundances of major phyla-level taxa for microbiomes from different sample types and stages along the farm-to-fork continuum of a pasture-raised broiler flock^1^, ^2^.

	**Hatchery (GIT) (%)**	**Brood (Feces) (%)**	**Pasture (Feces) (%)**	**Processing (SFR) (%)**	**Processing (Ceca) (%)**	**Processing (P-WCR) (%)**	**Final Product (FP-WCR) (%)**
Actinobacteria	1.39	4.16	6.32	5.49	3.50	1.11	1.60
Cyanobacteria	2.27	0.02	0.02	3.96	0.18	6.28	2.80
Firmicutes	10.20^B^	57.64^A^	68.26^A^	12.80^B^	61.34^A^	6.64^B^	16.73^B^
Proteobacteria	85.76^A^	28.72^B^	23.08^B^	74.25^A^	5.12^B^	84.81^A^	76.80^A^
Bacteroidetes	0.18^B^	7.96^B^	1.85^B^	2.85^B^	21.89^A^	0.70^B^	1.53^B^
Euryarchaeota	0.00^B^	0.04^B^	0.05^B^	0.02^B^	2.87^A^	0.00^B^	0.00^B^
Tenericutes	0.00^B^	0.40^B^	0.02^B^	0.05^B^	2.11^A^	0.05^B^	0.00^B^

1 Information in parentheses in the top row indicates the sample type (GIT, gastrointestinal tract; SFR, Skin & Feather Rinse; P-WCR, Processing Whole Carcass Rinse; FP-WCR, Final Product Whole Carcass Rinse).

2 *Superscript letters next to the a-diversity estimates indicated significantly different values for a single metric across a row, based on mean separation of ANOVA using p < 0.05 significance level*.

**Figure 5:**
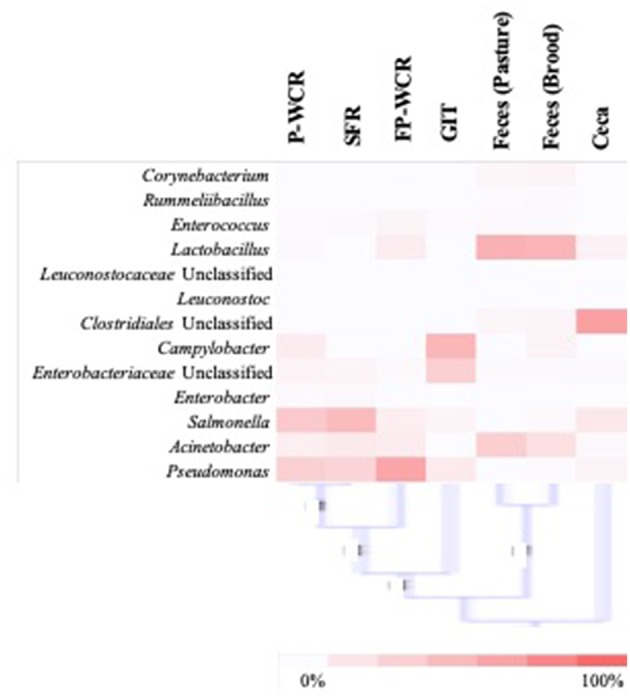
Analysis of the stringent core poultry-related microbiome, representing 13 taxa that were present in 75% of all samples along the farm-to-fork continuum of a pasture-raised broiler flock. The distribution of these taxa within each sample type/stage of the farm-to-fork continuum are shown by the heatmap (with higher concentrations denoted by darker red color), and WPGMA below the heatmap indicating the relatedness of the stringent core microbiomes within the different sample types/stages.

To simplify the genus-level taxa shifts throughout the farm-to-fork continuum (which contained 430 total taxa), the core poultry microbiome from all samples was determined. To accomplish this, OTUs that were present in 50 or 75% of all samples were identified. There were 105 taxa consistent across 50% core microbiome, and in most cases these OTUs were found in at least two sample types and/or stages throughout the farm-to-fork continuum (Table 2). The Hatchery samples did not possess any core OTUs unique to those GIT samples, whereas 20% of the core OTUs were unique to only the cecal samples. The only other stage to have >4% unique OTUs was the brood feces (11.1%). Refinement of the core microbiome focused on those OTUs that were present in at least 75% of all samples (Figure 5). The more stringent core microbiome consisted of 13 groups representing three phyla: Actinobacteria (*Corynebacterium*), Firmicutes (*Rummelbacillus, Enterococcus, Lactobacillus, Leuconostocaceae* unclassified, *Leuconostoc, Clostridiales* unclassified), and Proteobacteria (*Campylobacter, Enterobacteriaceae* unclassified*, Enterobacter, Salmonella, Acinetobacter, Pseudomonas*). Definite shifts in this core microbiome were observed throughout the farm-to-fork continuum, with the Firmicutes members being more prevalent in the fecal and cecal samples and the Proteobacteria being more abundant in the hatchery, processing, and final product samples (Table 3). The core microbiomes of the rinsate samples (SFR, P-WCR, FP-WCR) were more similar, with two rinsates collected during processing and the fecal core microbiomes from the brood and pasture identified as the most similar via WPGMA analyses (Figure 5). The only other longitudinal broiler microbiome study in the literature also detected *Corynebacterium, Lactobacillus, Campylobacter*, and *Enterobacter* in the core microbiome of fecal, litter, carcass rinse, and weep samples (23), although these samples were not collected from the same flock (pre-harvest, carcass rinse, and weep samples were all collected from different sources at different times). The difference in core microbiomes between that study and this one is likely due to the samples being collected from conventional-based poultry management systems.

**Table 2:** Shared (found in at least two sample types) and Unique (found in only one sample type) OTUs found within the core microbiome found in at least 50% of the different sample types and stages along the farm-to-fork continuum of a pasture-raised broiler flock^a^.

	**Shared OTUs (%)**	**Unique OTUs (%)**
Hatchery (GIT)	100.00	0.00
Brood (Feces)	87.60	11.10
Pasture (Feces)	93.40	4.00
Processing (SFR)	99.35	0.00
Processing (Ceca)	80.00	18.60
Processing (P-WCR)	99.10	0.72
Final Product (FP-WCR)	96.30	3.20

a *Information in parentheses in the first column indicates the sample type (GIT, gastrointestinal tract; SFR, Skin & Feather Rinse; P-WCR, Processing Whole Carcass Rinse; FP-WCR, Final Product Whole Carcass Rinse)*.

Table 3Relative abundances of the stringent core microbiome taxa (OTUs present in 75% of all samples) within the core and total microbiomes from different sample types and stages along the farm-to-fork continuum of a pasture-raised broiler flock^a^, ^b^.

**Table:** 

	**Hatchery (GIT)**		**Brood (Feces)**		**Pasture (Feces)**	
	**% Core^b^**	**% Total^c^**	**% Core**	**% Total**	**% Core**	**% Total**
*Corynebacterium*	0.00	0.00	5.65	1.39	4.26	3.11
*Rummelibacillus*	0.00	0.00	2.48	1.02	1.99	3.16
*Enterococcus*	1.59	1.27	2.54	0.87	2.97	5.87
*Lactobacillus*	0.00	0.00	48.57	31.62	51.46	39.86
*Leuconostocaceae* Unclassified	0.00	0.00	1.15	0.13	0.48	0.28
*Leuconostoc*	0.00	0.00	0.53	0.02	0.02	0.01
*Clostridiales* Unclassified	0.17	0.14	5.69	2.08	4.24	1.74
*Campylobacter*	48.26	41.12	6.31	0.06	0.48	0.21
*Enterobacteriaceae* Unclassified	30.39	23.61	2.67	0.57	2.09	1.21
*Enterobacter*	0.00	0.00	0.20	0.00	0.01	0.01
*Salmonella*	6.01	5.18	2.79	0.04	0.07	0.04
*Acinetobacter*	0.79	0.65	19.63	6.49	31.90	19.92
*Pseudomonas*	12.78	14.64	1.78	4.71	0.03	0.09
Total	100.00	86.61	100.00	49.00	100.00	75.50

**Table:** 

	**Processing (SFR)**		**Processing (Ceca)**		**Processing (P-WCR)**		**Final Product (FP-WCR)**	
	**% Core**	**% Total**	**% Core**	**% Total**	**% Core**	**% Total**	**% Core**	**% Total**
*Corynebacterium*	0.21	0.77	0.15	0.01	0.15	0.22	0.01	0.27
*Rummelibacillus*	0.04	0.03	0.27	0.03	0.21	0.18	0.86	0.69
*Enterococcus*	2.11	1.43	0.72	0.06	1.45	0.88	4.83	3.70
*Lactobacillus*	0.92	0.98	6.83	1.88	2.31	3.32	10.33	9.54
*Leuconostocaceae* Unclassified	0.02	0.01	0.19	0.01	0.01	0.01	0.02	0.01
*Leuconostoc*	0.01	0.01	0.13	0.01	0.01	0.00	0.01	0.01
*Clostridiales* Unclassified	0.50	0.24	62.96	6.12	0.01	0.01	0.09	0.07
*Campylobacter*	1.07	1.00	0.90	0.06	10.91	10.75	0.86	1.68
*Enterobacteriaceae* Unclassified	5.86	4.82	2.36	0.35	6.50	6.14	2.12	2.18
*Enterobacter*	4.05	4.04	1.24	0.21	3.01	3.29	0.64	0.59
*Salmonella*	44.68	35.09	14.80	2.15	35.63	31.07	9.59	7.38
*Acinetobacter*	12.93	8.13	3.30	0.22	9.85	7.86	11.09	9.64
*Pseudomonas*	27.60	16.22	6.16	0.45	29.96	21.39	59.55	47.51
Total	100.00	72.79	100.00	11.57	100.00	85.12	100.00	83.28

a Information in parentheses in the top row indicates the sample type (GIT, gastrointestinal tract; SFR, Skin & Feather Rinse; P-WCR, Processing Whole Carcass Rinse; FP-WCR, Final Product Whole Carcass Rinse).

b Represents the relative abundance of each taxa within the stringent core microbiome including OTUs present in 75% of all samples (13 total taxa).

c *Represents the relative abundance of each taxa within the total microbiome without excluding OTUs based on presence in a set percentage of samples (430 taxa)*.

The WPGMA findings align with what was observed for the total microbiomes (Figure 2), and the cecal microbiomes were found to be very unique compared to all other microbiomes. The 13 taxa of the 75% core microbiome represented ~50% or more of the total microbiome of the other six stages per sample types, representing an average of ~75% of the total microbiome. However, these 13 taxa only accounted for ~12% of the total cecal microbiome, making this microbiome more unique (Table 3). In combination with the fact that 50% core microbiomes contained 20% unique OTUs, these results demonstrate the very unique bacterial communities contained within this part of the poultry GIT. Other studies based on conventionally grown birds have demonstrated that cecal microbiomes are unique from other poultry-related microbiomes collected from the farm (23, 61) or from other sections of the gastrointestinal tract (24, 62). Considering the cecum is a common sample target for food safety research, this data suggests that the uniqueness of ceca microbial ecology needs to be considered. The survival and persistence of potential pathogens within the very unique cecal environment may not correlate with pathogen survival in different microbial communities throughout the farm-to-fork continuum (Figures 2, 5). According to the data from the current study, it is possible that post-processing carcass rinses (P-WCR) may represent a better proxy for what is found on the final product (FP-WCR) compared to the ceca.

### Specific Focus on Foodborne Pathogens

**Figure 6:**
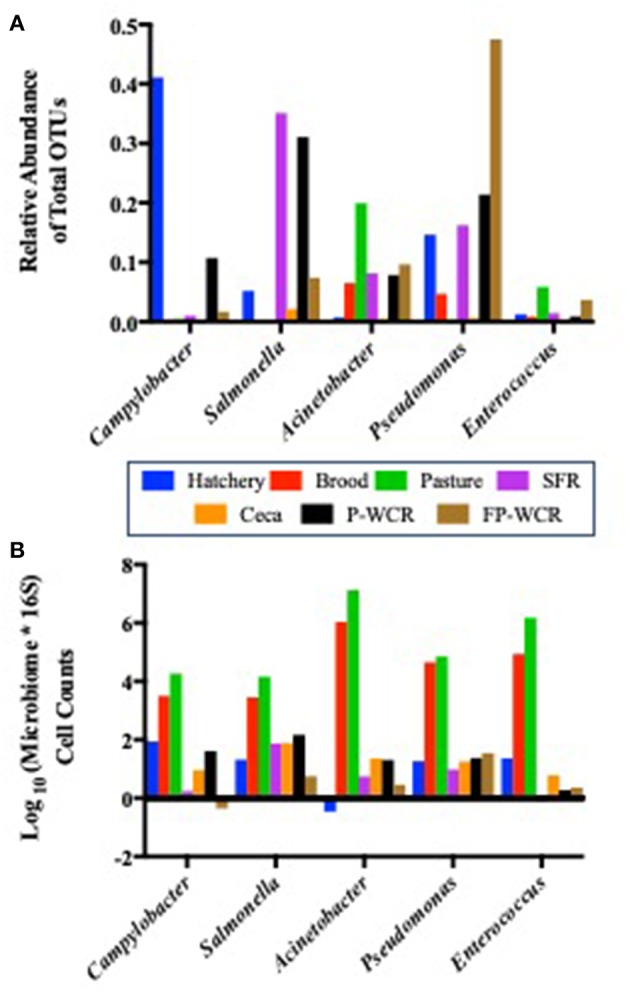
Prevalence of five core zoonotic taxa within the total microbiomes from different sample types and stages along the farm-to-fork continuum of a pasture-raised broiler flock. **(A)** Relative abundances of the five core zoonotic taxa within the total microbiomes (430 total taxa). **(B)** Log_10_-transformed quantified microbiome cell counts of the five core zoonotic taxa, based on multiplying the microbiome relative abundance data by the total bacterial counts for each sample according to 16S qPCR analysis.

Due to the increased access to the environment and other farm animals in the pastured poultry management system (32, 63), there is a hypothesis that this exposure would increase the prevalence of foodborne pathogens within pasture-raised flocks. One of the most interesting outcomes of the stringent core microbiome analysis above was the inclusion of known foodborne pathogens (*Campylobacter, Salmonella*) and genera that could potentially possess foodborne pathogen species (*Pseudomonas, Enterococcus*) or considered emerging pathogens (*Acinetobacter*). While much of the food safety-related work in poultry is focused on the post-harvest environments (processing, final product), the above data (Figure 5, Table 3) demonstrates that these zoonotic bacterial pathogens are consistent members of the poultry microbiome. The persistence of these pathogens are consistent from the pre-hatch through the post-processing environments to the consumer's kitchens. Therefore, these five foodborne pathogen taxa within the total microbiomes were specifically investigated throughout the entire lifetime of this pasture-raised broiler flock (Figure 6).

While each of these taxa were present in all stages and/or sample types, their relative abundance within the total microbiomes of each stage and/or sample type shifted dramatically (Figure 6A). The most abundant zoonotic taxa in total microbiomes for each stage of the farm-to-fork continuum were: *Campylobacter* in the hatchery GIT samples (41.1%); *Acinetobacter* in the brood and pasture fecal samples (6.5 and 19.9%, respectively); *Salmonella* in the processing SFR, ceca, and P-WCR samples (35.1, 2.1, 31.1%, respectively); and *Pseudomonas* in the FP-WCR samples (47.5%). *Enterococcus* was present throughout the study but was never the dominant zoonotic taxa and always represented < 0.5% of the total microbiomes for any sample. Finding these known or potential foodborne pathogens as endemic taxa within the core poultry-related microbiome has definite implications for the use of future intervention strategies to reduce these zoonotic populations. Focusing on a single stage of the farm-to-fork continuum (typically the processing environment) may only result in a partially efficacious intervention, since these pathogens can thrive at all stages of the management system. These findings, while they do not indicate virulence, do highlight the need to take a more systems-based approach to intervention strategies that look deeper into the dynamics of the specific zoonotic serotypes/species throughout the farm-to-fork continuum to determine whether broader or more targeted strategies are needed, and at what stage they are going to be most effective.

While microbiome analysis allows for an assessment of bacterial communities and its individual members, it only does so pseudo-quantitatively by determining each taxa's relative abundance to the whole bacterial population within those samples. This must be considered when looking at the data within Figure 6A, where known poultry pathogens such as *Campylobacter* and *Salmonella* represent 1.7 and 7.4% of the final product microbiomes, respectively, but only 0.04 and 0.06% of the fecal microbiomes. Therefore, qPCRs were performed to quantify the total bacterial populations in the farm-to-fork continuum samples, as well as specific foodborne pathogens (*Salmonella, C. jejuni*, and *L. monocytogenes*; Table 4).

**Table 4:** qPCR quantification (log_10_-transformed) of total bacteria and foodborne pathogens (*Salmonella, Campylobacter jejuni, Listeria monocytogenes*) from different sample types and stages along the farm-to-fork continuum of a pasture-raised broiler flock^a, b^.

	**Pre-harvest**			**Post-harvest**			
	**Hatchery**	**Brood**	**Pasture**	**Processing**			**Final Product**
**Target (gene)**	**GIT**	**Feces**	**Feces**	**SFR**	**Ceca**	**P-WCR**	**FP-WCR**
Total Bacteria (16S)	2.39 ± 1.72	6.72 ± 0.66	7.34 ± 0.46	1.61 ± 0.94	3.89 ± 0.70	2.11 ± 0.73	1.68 ± 0.50
*Salmonella* spp. (invA)	0.10 ± 0.12	0.24 ± 0.13	0.22 ± 0.34	0.42 ± 0.15	0.52 ± 0.10	0.00 ± 0.00	0.00 ± 0.00
*Campylobacter jejuni* (hipO)	1.43 ± 0.21	1.72 ± 0.56	1.74 ± 0.95	0.16 ± 0.23	0.11 ± 0.35	1.30 ± 1.21	0.00 ± 0.00
*Listeria monocytogenes* (hylA)	0.19 ± 0.40	0.17 ± 0.33	0.73 ± 0.82	0.00 ± 0.00	0.00 ± 0.00	0.00 ± 0.00	0.00 ± 0.00

a Information in the third row indicates the sample type (GIT, gastrointestinal tract; SFR, Skin & Feather Rinse; P-WCR, Processing Whole Carcass Rinse; FP-WCR, Final Product Whole Carcass Rinse).

b *Values represent the average ± standard deviation for replicate samples for each sample type (N = 12, 5, 10, 12, 12, and 3 for the GIT, Brood Feces, Pasture Feces, SFR, Ceca, P-WCR, and FP-WCR, respectively)*.

To determine if the *Campylobacter* and *Salmonella* are numerically more abundant in the final product or just in the FP-WCR samples, the total bacterial populations for each sample were determined by 16S qPCR (40). Using the qPCR C_T_ values (16S copies per PCR) and the relatively abundance values from the microbiome data, a cell count was determined (Figure 6B). The quantification of the microbiome data shows that all five pathogens taxa within the core microbiome were significantly (*p* < 0.001) higher in the brood and fecal samples compared to all hatchery, processing, and final product samples. For all taxa, the calculated cell count was lower than 1 log in the FP-WCR samples (brown bar). This shift in increased prevalence in the fecal samples is directly due to the fact that the brood and pasture fecal samples possessed much larger bacterial densities (6.7 and 7.3 log_10_ 16S copies/qPCR, respectively) compared to the hatchery (2.6 log_10_ 16S copies/qPCR), processing (1.6, 3.9, 2.1 log_10_ 16S copies/qPCR for SFR, ceca, and P-WCR, respectively), and final product samples (1.7 log_10_ 16S copies/qPCR). It appears that while *Campylobacter* and *Salmonella* represent a larger portion of the FP-WCR microbiome, numerically the populations were ~3 logs higher in the fecal samples than in the final product samples. There are numerous potential biases with this quantification of the microbiome data, including the use of different 16S rDNA primer sets for the qPCR and microbiome data. Additionally, the 16S copy number can range from 1 to 7 depending on the bacteria, which can also bias the data; however, that bias should be relatively equal for all samples and the resultant trends should be accurate.

Since all five pathogenic taxa within the core microbiome were more prevalent in the fecal samples, correlations to physiochemical (pH, EC, moisture), and nutrient data (total C, total N, C:N ratio, elements) were performed to determine any potential environmental drivers of their relative abundances within these fecal microbiomes (Table 5). Just under half (12 of 25) of the measured environmental variables showed significant correlation to relative abundances of any of the pathogenic core taxa. Of the five zoonotic core taxa, two were not significantly correlated to any measured environmental variables (*Salmonella, Pseudomonas*), while *Campylobacter* and *Enterococcus* showed a significant correlation to only C:N ratio. Previous poultry-related microbiome work has not shown any associations between *Campylobacter* to other bacterial microbiome taxa (23), but this current study shows that C:N ratio is significantly correlated to not only *Campylobacter* and *Enterococcus*, but also was shown to have a significant effect of the β-diversity distribution of the total fecal microbiomes (Figure 3). Additionally, *Acinetobacter* was significantly correlated to 11 of the 25 environmental variables, with *R*^2^ values ranging from 0.333 to 0.521. These data provide insight into potential physiochemical variables that effect foodborne pathogen abundance during the on-farm production stage of pastured broiler management, which could potentially lead to the development of pre-harvest mitigation strategies if these parameters can be modulated in the broiler gut.

**Table 5:** Correlation of pathogenic taxa within the stringent core microbiome (OTUs present in 75% of all samples) to elemental concentrations within pre-harvest (brood, pasture) fecal samples of a pasture-raised broiler flock^a, b, c^.

	**Total C (%)**	**Total N (%)**	**C:N Ratio**	**Ca (ppm)**	**K (ppm)**	**Mg (ppm)**	**Mn (ppm)**	**Na (ppm)**	**P (ppm)**	**S (ppm)**	**Si (ppm)**	**Zn (ppm)**
*Campylobacter*	0.627	0.464	**0.0172** (*0.486*)	0.355	0.554	0.541	0.665	0.321	0.641	0.575	0.646	0.616
*Salmonella*	0.407	0.447	0.355	0.160	0.277	0.312	0.469	0.176	0.398	0.285	0.218	0.431
*Acinetobacter*	**0.0122** (*0.521*)	**0.0184** (*0.479*)	0.653	**0.0196** (*0.435*)	**0.009** (*0.510*)	**0.0152** (*0.461*)	**0.0496** (*0.333*)	**0.0188** (*0.440*)	**0.044** (*0.347*)	**0.0162** (*0.454*)	**0.0357** (*0.370*)	**0.0396** (*0.359*)
*Pseudomonas*	0.549	0.490	0.463	0.662	0.600	0.767	0.866	0.422	0.775	0.498	0.892	0.866
*Enterococcus*	0.548	0.341	**0.0428** (*0.382*)	0.705	0.434	0.556	0.496	0.291	0.510	0.564	0.955	0.389

a Physiochemical and nutrient variables that did not have any significant correlations to bacterial taxa are not included in this table.

b Information in parentheses in the top row indicates units of concentration per gram of feces.

c *Values represent p-values of the correlation analysis, with the significant correlations (p < 0.05) bolded. The R^2^ values for the significant correlations are provided in italics within parentheses below the p-value*.

Multiple studies have shown that alternative poultry production management systems, including pasture-raised, can reduce *Salmonella* prevalence compared to conventional systems in pre-harvest samples (55, 64), but results can vary in the post-harvest environments (65–68). *Campylobacter* was typically the most prevalent of the zoonotic pathogens recovered from alternative poultry management systems, with *Campylobacter* found within various pre-harvest and post-harvest/final product samples (54, 55, 66–68), with counts higher in farm samples but prevalence being higher in processing/final product samples (23, 56). Both of these pathogens represented important members of the farm-to-fork core microbiome of this current study, and their abundance varied along the farm-to-fork continuum. Having higher pre-harvest counts, but higher prevalence in post-harvest samples has been previously reported for *Campylobacter* in conventional (23) and pastured poultry management systems (56), although no data was available for *Salmonella* or the other three zoonotic core taxa from this study. Given the importance of *Acinetobacter* species as a potentially drug resistance pathogen in clinical settings (12), it is important to further elucidate the environmental drivers of *Acinetobacter* relative abundance within the broiler farm environment, since it is a relatively uninvestigated reservoir for this emerging pathogen.

## Conclusions

While there are significant shifts in the poultry microbiome from the pre-hatch to the final product stage, there was a core microbiome that was present throughout the entire farm-to-fork continuum of this pastured-raised broiler flock. Investigations of these microbiomes revealed several important findings that need to be further investigated, including (1) the relatively rapid (by 1 week of age) development of stable gut microbiome in these broilers, as evidenced by the fecal microbiomes; (2) the uniqueness of the cecal microbiome and the cecal environment and how poorly it correlates to the final product microbiome (and what implications that may have for food safety-related research); and (3) the presence of known pathogens (*Salmonella, Campylobacter*) and potential/emerging pathogens (*Pseudomonas, Enterococcus, Acinetobacter*) in the core microbiome found throughout the farm-to-fork continuum, which underlines the importance of understanding these pathogens from a longitudinal pre-harvest and post-harvest perspective. It should be noted that these results are only from a single small pasture-raised flock, but on-going research has expanded to 10 more farms and 40 more flocks over 4 years, and preliminary assessments of the data support the three major findings presented above. Therefore, these findings demonstrate the need for further longitudinal, farm-to-fork studies to understand the ecology of these bacteria to develop better abatement/intervention strategies to improve animal, environmental, and public health in alternative, as well as conventional, poultry production systems.

## Ethics Statement

Live animals were managed and handled solely by the collaborating farmer, including the butchering process. Laboratory technicians only handled environmental samples (feces, soil) and poultry samples post-mortem (ceca, rinses).

## Author Contributions

MR designed and executed the study, analyzed the data, and wrote the manuscript. AL, AC, and JG helped in the analysis of the microbiome (AL, AC) and pathogen specific (JG) data. KH assisted in the initial design of the experiment. SR and KF assisted in the construction and revision of the manuscript and provided analytical support for the microbiome analysis.

### Conflict of Interest Statement

The authors declare that the research was conducted in the absence of any commercial or financial relationships that could be construed as a potential conflict of interest. The handling editor declared a shared affiliation, though no other collaboration, with the authors MR, AL, and JG.
